# Dr. John W. Burchard

**Published:** 1912-11

**Authors:** 


### OBITUARIES.

Dr. John W. Burchard, Buffalo, 1884, died at his home in Bradley, South Dakota, August 15, from pneumonia, aged 65.

